# Antimicrobial Resistance: A Bibliometric Review of Patient Health, Mechanisms, and Therapeutic Strategies

**DOI:** 10.3390/pathogens15030288

**Published:** 2026-03-06

**Authors:** Peter E. Murray, Jonathan A. Coffman, Franklin Garciá-Godoy

**Affiliations:** 1 Independent Researcher, San Francisco, CA 94102, USA; 2 Department of Biomedical Sciences, Mercer University School of Medicine, Mercer, GA 31207, USA; 3 College of Dentistry, University of Tennessee Health Science Center, Memphis, TN 38103, USA; fgarciagodoy@gmail.com

**Keywords:** infectious diseases, viruses, fungi, parasites, antibiotics, antibacterial, hygiene

## Abstract

Antimicrobial resistance is a growing global health crisis, with projections estimating up to 10 million deaths annually and more than 130 million hospitalizations attributable to resistant infections. Resistance emerges through microbial adaptation to sustained antimicrobial pressure, resulting in genetic and phenotypic mechanisms such as target mutations, enzymatic drug inactivation, efflux pump activation, biofilm formation, and metabolic adaptation that drive the emergence of multidrug-resistant pathogens and limit effective therapy. This bibliometric review analyzes the MEDLINE-indexed articles to characterize patient health and age factors, resistance mechanisms, pathogen types, and therapeutic strategies associated with antimicrobial resistance. The findings demonstrate that antimicrobial resistance is disproportionately reported among adults and chemotherapy-treated patients, with transmitted resistance emerging as the dominant driver, emerging from antibiotic misuse or overuse. Despite extensive mechanistic knowledge, treatment remains largely focused on escalation to newer antibiotics, while combination and adjunctive therapies are less commonly reported. These results analyze 308,290 studies to identify patient-related contexts, resistance mechanisms, pathogens, and therapeutic strategies.

## 1. Introduction

Antimicrobial resistance has become one of the most urgent and complex threats facing modern medicine and global public health [1]. An estimated 130 million hospitalizations and more than 10 million deaths worldwide are projected to be attributable to resistant infections, many of which could be mitigated through the development of more effective antimicrobial therapies [2]. Beyond its direct toll on morbidity and mortality, antimicrobial resistance is driving escalating healthcare costs and steadily eroding the foundations of modern clinical practice [3,4].

Human-driven factors play a central role in the emergence and spread of antimicrobial resistance. Resistance is accelerated by the overuse, misuse, and underuse of antibiotics, as well as by exposure to antimicrobial compounds in cosmetics, heavy metals, disinfectants, and inadequate sanitation and hygiene in community and healthcare settings [5]. These pressures are compounded by environmental contributors, including the natural presence of resistance genes, improper disposal of unused antimicrobials, contamination from wildlife, agricultural practices, and pharmaceutical waste, and the continual introduction of spontaneous genetic mutations [6,7].

Antimicrobial resistance arises when pathogenic microorganisms acquire genetic mutations and adaptive cellular mechanisms that enable them to survive exposure to antimicrobial agents and disinfectants [8]. This evolutionary process is driven by sustained selective pressure from widespread antimicrobial use and repeated disinfectant exposure, allowing bacteria, viruses, fungi, and parasites to persist, despite treatments that would normally eliminate them [9,10]. Multidrug-resistant infections are associated with higher rates of treatment failure, prolonged illness, and increased mortality, often necessitating more aggressive, toxic, and costly therapeutic interventions that further strain patients and healthcare systems alike [11].

The development of antimicrobial resistance is fundamentally driven by microbial evolution under sustained selective pressure [12]. Bacteria, viruses, fungi, and parasites exhibit extraordinary genetic plasticity, enabling rapid adaptation to the hostile environments created by antimicrobial exposure [13]. Through spontaneous mutation, horizontal gene transfer, and epigenetic regulation, pathogens acquire traits that diminish drug susceptibility or neutralize therapeutic activity altogether [14]. These adaptations include alteration of antimicrobial targets, enzymatic degradation or modification of drugs, reduced membrane permeability, active efflux of antimicrobial agents, and the formation of protective biofilms that insulate microbial communities from treatment [15]. Pathogens may express target protection proteins that shield essential enzymes from drug binding, alter metabolic pathways to bypass inhibited steps, or reorganize cellular structures to reduce drug penetration [16]. Efflux pump systems can expel multiple classes of antibiotics simultaneously, conferring broad-spectrum resistance with minimal fitness cost [17]. Enzymatic mechanisms such as beta-lactamases and carbapenemases continue to diversify, eroding the efficacy of drugs once considered resilient to resistance [18,19]. As these mechanisms accumulate within individual organisms and disseminate across populations, they give rise to multidrug-resistant and extensively drug-resistant strains that are increasingly difficult to control [20] and, in some cases, nearly impossible to eradicate.

Among the most prevalent and clinically significant resistant infections are those caused by Methicillin-resistant *Staphylococcus aureus* (MRSA), a pathogen responsible for a wide spectrum of disease, including skin and soft tissue infections, surgical site and wound infections, osteomyelitis, pneumonia, and life-threatening bloodstream infections [21,22,23].

Treatment of MRSA infections depends on the site and severity of disease, as well as patient-specific factors, such as immune status and renal function [24,25,26,27]. 

Beyond MRSA, resistant infections caused by organisms such as vancomycin-resistant *Enterococcus* [28], carbapenem-resistant *Enterobacterales* [29], and multidrug-resistant *P. aeruginosa* [30] present equally formidable challenges. These pathogens are frequently associated with limited therapeutic options, delayed effective treatment, and higher rates of treatment failure. Collectively, they illustrate how antimicrobial resistance narrows the scope of effective therapy, prolongs hospital stays, and substantially increases patient morbidity and mortality, underscoring the critical need for enhanced antimicrobial strategies and expanded research to address this escalating threat.

Clinically, resistant infections frequently present with non-specific or atypical symptoms, delaying diagnosis and complicating the initiation of appropriate therapy [31]. Persistent fever, ongoing inflammation, treatment failure, and recurrent infection are common clinical features, often prompting repeated or prolonged courses of broad-spectrum antimicrobials that further intensify selective pressure for resistance [32]. In severe cases, resistant infections may progress to sepsis, multi-organ dysfunction, or death, underscoring the critical importance of early recognition and timely, targeted intervention [33]. As resistance becomes more prevalent, empirical treatment strategies are increasingly unreliable, forcing clinicians to rely on last-line agents that are often less effective, more toxic, or limited by cost and availability [34].

The clinical burden of antimicrobial resistance is controversial but is generally not evenly distributed across patient populations [35]. Susceptibility to resistant infection is shaped by a combination of age, immune status, comorbid disease, prior antimicrobial exposure, and frequency of healthcare contact [36]. Older adults and immunocompromised individuals, including patients with malignancy, organ transplants, autoimmune disorders, or chronic inflammatory conditions, are particularly vulnerable due to impaired host defenses and repeated exposure to healthcare environments where resistant organisms are prevalent [37]. Infants and young children also face elevated risk, as immature immune systems and restricted therapeutic options limit effective treatment choices [38]. Understanding how patient-level variables intersect with microbial resistance mechanisms is essential for identifying high-risk populations and developing targeted prevention and management strategies [39,40,41,42].

Despite extensive research activity in antimicrobial resistance, the existing literature remains fragmented across disciplines, organisms, patient populations, and therapeutic approaches [40,41]. These gaps hinder the development of effective prevention strategies and delay the implementation of targeted interventions for populations most likely to experience disability or mortality from difficult-to-treat or untreatable infections [42].

Bibliometric review methodologies [43] offer a powerful framework for synthesizing this expansive and heterogeneous body of research [44]. By quantitatively and qualitatively examining publication trends, study designs, and thematic focus areas, bibliometric analyses can identify dominant research trajectories, emerging priorities, and persistent knowledge gaps within the field [45]. When integrated with systematic evaluation of patient characteristics, resistance mechanisms, and therapeutic outcomes, this approach [43,44,45] could provide a comprehensive perspective on the current state of antimicrobial resistance research and clarify directions for future investigation.

Accordingly, the purpose of this bibliometric review is to comprehensively analyze the MEDLINE literature database [43], thereby mitigating the selection bias that often characterizes traditional meta-analyses and systematic reviews reliant on narrowly defined PRISMA inclusion criteria [46]. This methodology enables a broad, integrative appraisal of the scientific and clinical literature on antimicrobial resistance. The aim of this study is to descriptively examine how antimicrobial resistance is addressed in MEDLINE-indexed publications, with respect to reported patient-related contexts, resistance mechanisms, pathogens, and therapeutic strategies.

## 2. Materials and Methods

This bibliometric analysis was conducted in accordance with established methodological guidelines for frequency and distribution-based bibliometric research [43,44,45]. This study is a descriptive analysis at the publication level based on the frequency and thematic distribution of MEDLINE articles. A comprehensive search of the PubMed MEDLINE database was performed over the period from 20 December 2025, to 8 January 2026. Thus, all the studies published from 1945 to 2026 were included, and none were excluded. The search strategy centered on the primary term “antimicrobial resistance” and was expanded using additional relevant keywords, as detailed in the accompanying figures. All studies indexed in MEDLINE under the term “antimicrobial resistance” were included, while studies not meeting this criterion were excluded from the search. There was no manual exclusion of studies by the investigators. This methodology assumes that studies indexed under the selected search terms were classified accurately by the database and that any indexing inaccuracies or inclusion of irrelevant records, such as non-human studies or editorials, were randomly distributed across the dataset and, therefore, unlikely to materially influence overall frequency and distribution-based bibliometric patterns.

No bibliometric software was used; the MEDLINE results were transferred into an Excel (Microsoft, Redmond, WA, USA) worksheet for bibliometric and statistical analysis and also pie chart rendering.

To minimize selection bias and ensure comprehensive coverage of the literature, all retrieved records were included in the analysis using a previously published bibliometric methodology [43,44,45]. No study-level exclusions or filtering criteria were applied. Descriptive and comparative statistical analyses [43,44,45] were conducted to assess the volume, distribution, and characteristics of antimicrobial resistance-related publications across predefined categories. Because this approach did not involve the selective inclusion or exclusion of studies by the investigators, a formal PRISMA-based risk of bias assessment was not required [46].

Comparisons between publication groups [43] were evaluated using chi-squared (χ^2^) tests [47]. Statistical significance was defined as a *p*-value of less than 0.05.

## 3. Results

### 3.1. Accelerating Growth of Research Publications in Antimicrobial Resistance

The MEDLINE search indexed under the term “antimicrobial resistance” identified up to 308,290 studies of all types, including epidemiological studies and scientific research; however, the total number of studies included in each analysis varied according to the applied search criteria, as detailed in the Supplemental Data.

**Figure 1:**
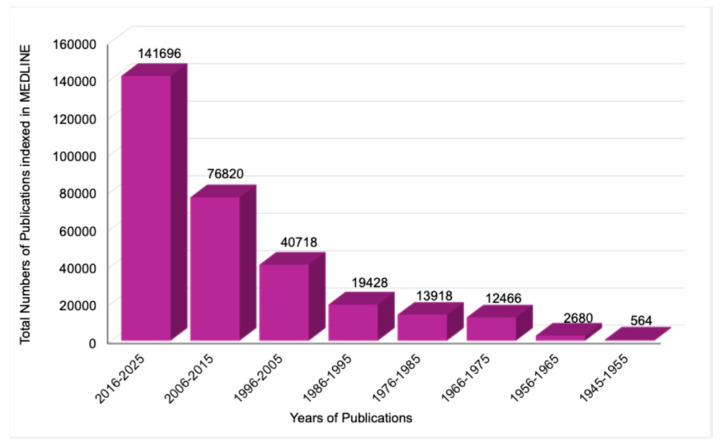
Accelerating growth of research publications in antimicrobial resistance. This bar chart illustrates the distribution of 308,290 antimicrobial resistance publications indexed in MEDLINE by decade, spanning from 1945 through 2025.

Notably, nearly half of all publications on antimicrobial resistance have been published within the past decade, from 2016 to 2025, although relevant studies have been indexed as far back as 1945. This pattern demonstrates a marked and accelerating growth in global research output, consistent with an exponential expansion of scientific attention to antimicrobial resistance in recent years. This distribution was statistically significant (χ^2^ = 427,928, *p* < 0.0001), as illustrated in Figure 1.

A log-linear regression of MEDLINE-indexed publication counts by year demonstrated that studies indexed under the term “antimicrobial resistance” have increased exponentially over time, consistent with the following model:

N(y) = 39.5 · e^(0.0837 · (y − 1945))

where

N(y) is the number of publications in year y;
1945 is the baseline year of the dataset;
0.0837 (8.37%) is the estimated annual growth rate.

### 3.2. Antimicrobial Resistance by Age Group

The relationship between patient age groups and antimicrobial resistance is controversial, with some studies suggesting a disproportionate burden among children [47], while others report higher prevalence in elderly populations [48].

In contrast to these prior conflicting publications [47,48], the present analysis demonstrates a clear age-related distribution of antimicrobial resistance. 

**Figure 2:**
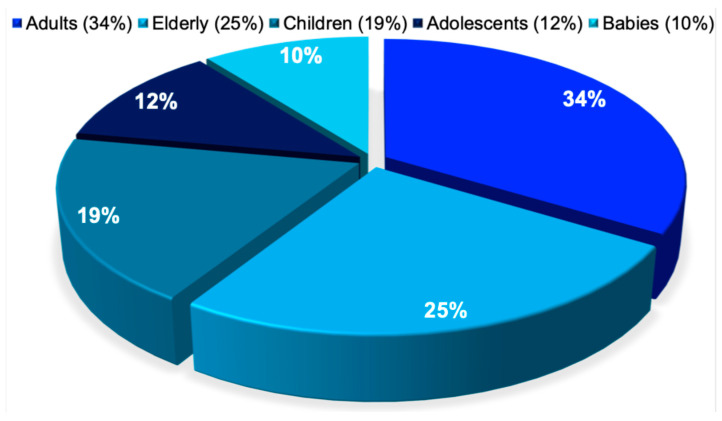
Antimicrobial resistance by age group. This pie chart depicts the age distribution of antimicrobial resistance cases reported across 134,835 publications, classified into the following groups as defined by the respective study authors: adults aged 18 to 64 years, elderly individuals older than 65 years, children aged 1 to 9 years, adolescents aged 10 to 17 years, and infants younger than 1 year.

We included MEDLINE-indexed studies that stratified the age group of the patients using the following keywords: adults, elderly, adolescents, children, and babies. The adults accounted for approximately one third of affected patients (34%), followed by elderly adults (25%), children (19%), adolescents (12%), and babies, who represented the smallest proportion (10%). This distribution was statistically significant (χ^2^ = 70.16, *p* < 0.0001), as illustrated in Figure 2.

### 3.3. Antimicrobial Resistance Reported in Immunocompromised Patients

The association between specific immunocompromising conditions and antimicrobial resistance has been reported inconsistently in the literature, with some studies identifying human immunodeficiency virus infection or AIDS as the most heavily affected population [49], while others emphasize solid organ transplant recipients as bearing the greatest burden [50].

**Figure 3:**
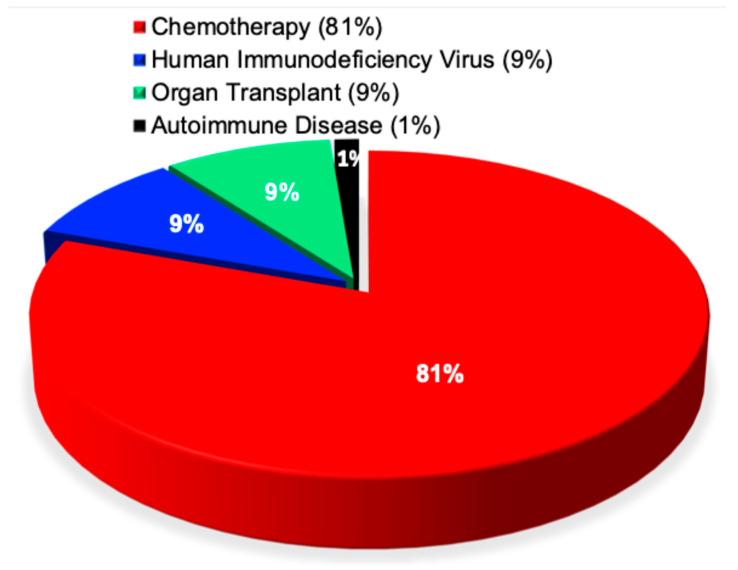
Antimicrobial resistance reported in immunocompromised patients. This pie chart shows the proportions of antimicrobial resistance reported across 2725 publications including adults (38%), elderly (30%), children (16%), adolescents (9%), and babies (7%) who have chemotherapy, human immunodeficiency virus, organ transplant, and autoimmune disease.

In contrast to these divergent findings [49,50], the present analysis reveals a distinct distribution of antimicrobial resistance across immunocompromised patient groups. Individuals undergoing chemotherapy accounted for the vast majority of resistant infection cases, representing approximately 81% of immunocompromised patients affected by antimicrobial resistance. This was followed by patients with HIV AIDS (9%), organ transplant recipients (9%), and those with autoimmune diseases, who comprised less than 1% of cases. This distribution was highly statistically significant (χ^2^ = 4294, *p* < 0.0001), as shown in Figure 3.

### 3.4. Antimicrobial Resistance in Patients with Chronic Disease

The reasons for chronic antimicrobial resistance are multifactorial and variably emphasized across the literature. Some studies report a disproportionate burden among patients with cancer [51], while others identify drug-resistant infections themselves as the primary contributor to chronic resistance [52].

**Figure 4:**
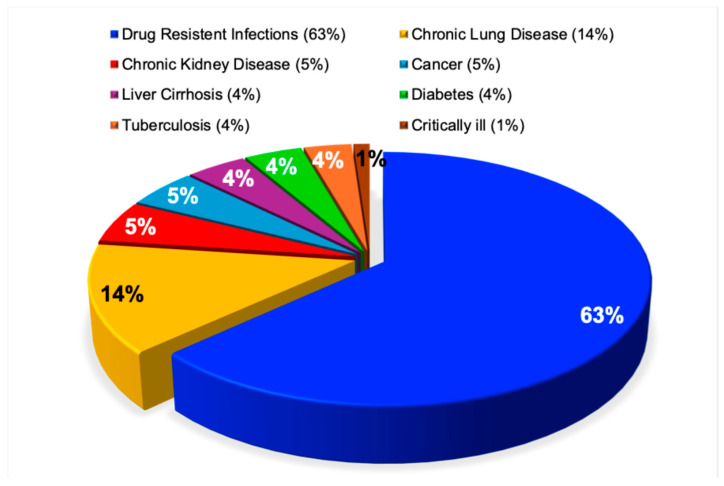
Antimicrobial resistance in patients with chronic disease. This pie chart shows the proportions of antimicrobial resistance reported across 8647 publications including adults (42%), elderly (35%), children (9%), adolescents (10%), and babies (3%) who have drug-resistant infections and chronic conditions, including lung disease, kidney disease, and cancer.

In contrast to these heterogeneous findings [51,52], the present analysis reveals a clear distribution of chronic disease factors associated with antimicrobial resistance. Patients suffering from drug-resistant infections accounted for the majority (63%) of chronic cases. Patients suffering from chronic lung disease were the next most common contributor (14%), followed by chronic kidney disease, cancer, liver cirrhosis, diabetes, and tuberculosis, each accounting for approximately 4% to 5% of cases. Critically ill patients represented the smallest proportion, comprising less than 1% of reported cases. This distribution was highly statistically significant (χ^2^ = 23,344, *p* < 0.0001), as illustrated in Figure 4.

### 3.5. Research on the Mechanisms Driving the Development of Antimicrobial Resistance

Research into the reasons for developing antimicrobial resistance has been reported inconsistently across the literature. Some studies identify antibiotic overuse as the primary driving force behind resistance development [53], whereas others emphasize the dominant role of transmitted resistance [54] or inadequate hygiene and infection control practices in facilitating the emergence and spread of resistant organisms [55].

**Figure 5:**
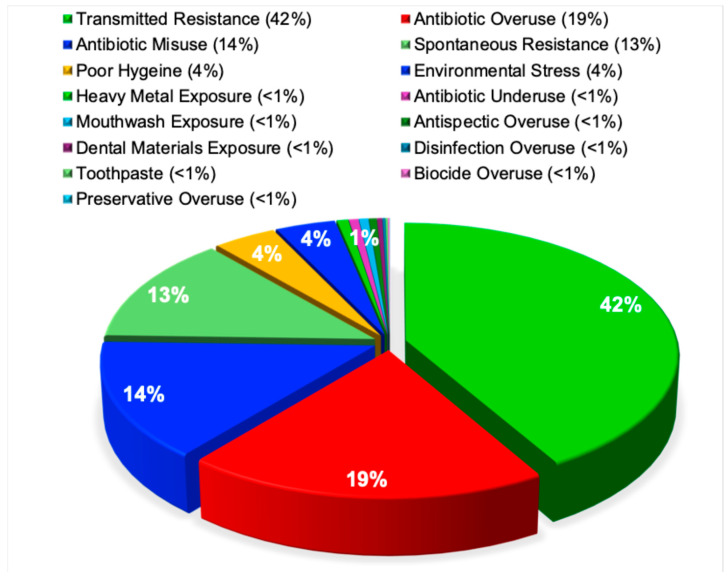
Research on the mechanisms driving the development of antimicrobial resistance. This pie chart shows the proportions of 3894 research publications on the mechanisms driving the development of antimicrobial resistance, including transmitted resistance, antibiotic overuse and misuse, spontaneous resistance, and hygiene-related factors.

In contrast to these divergent conclusions [53,54,55], the present analysis reveals a distinct and statistically robust hierarchy of factors associated with the development of antimicrobial resistance. Transmitted antimicrobial resistance accounted for the largest proportion of research (42%), underscoring the central role of person-to-person and environmental transmission. This was followed by antibiotic overuse (19%), antibiotic misuse (14%), and spontaneous resistance arising from genetic mutation (13%). Poor hygiene and environmental stressors each contributed approximately 4% of cases. All remaining factors individually accounted for fewer than 1% of cases, including heavy metal exposure, antibiotic underuse, exposure to mouthwashes or dental materials, antiseptic or disinfectant overuse, toothpaste, biocide overuse, and preservative overuse. This distribution was highly statistically significant (χ^2^ = 10,852, *p* < 0.0001), as illustrated in Figure 5.

### 3.6. Antimicrobial Resistance by Patient Health Status

Clinical manifestations of antimicrobial resistance have been described inconsistently in the literature. Some studies characterize the resistant infection itself as the primary clinical feature [56], while others emphasize specific symptoms, such as fever [57] or pain [58].

**Figure 6:**
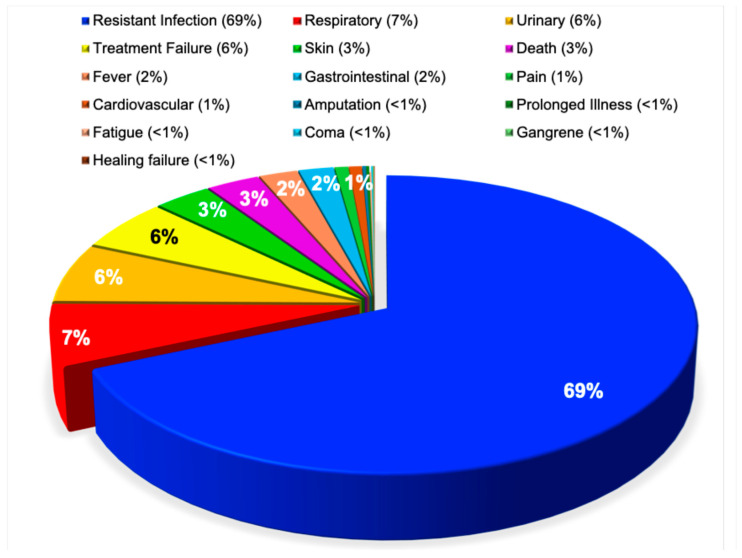
Antimicrobial resistance by patient health status. This pie chart illustrates the distribution of 103,622 research publications on antimicrobial resistance, stratified by patient health status, including studies involving resistant infections, respiratory tract infections, and urinary tract infections.

In contrast to these heterogeneous reports [56,57,58], the present analysis demonstrates a distinct distribution of patient health statuses associated with antimicrobial resistance. Overt resistant infection was the most frequently reported manifestation, accounting for 69% of cases. This was followed by respiratory symptoms (7%), urinary symptoms (6%), and treatment failure (6%). Cutaneous manifestations, death, fever, and gastrointestinal symptoms each accounted for approximately 2% to 3% of cases. All other patient health statuses, including pain, cardiovascular complications, amputation, prolonged illness, fatigue, coma, gangrene, and impaired wound healing, were reported in fewer than 1% of cases. This distribution was highly statistically significant (χ^2^ = 698,855, *p* < 0.0001), as illustrated in Figure 6.

### 3.7. Research into Treatments for Antimicrobial Resistance

Clinical approaches to the management of antimicrobial resistance are reported inconsistently across the literature. Some studies describe antibiotic combination therapy as the predominant strategy [59], whereas others emphasize escalation to higher potency or higher dose antibiotics [60], surgical intervention [61], or alternative modalities, such as photodynamic therapy [62]. This heterogeneity reflects both differences in study populations and the absence of a unified therapeutic framework for resistant infections.

**Figure 7:**
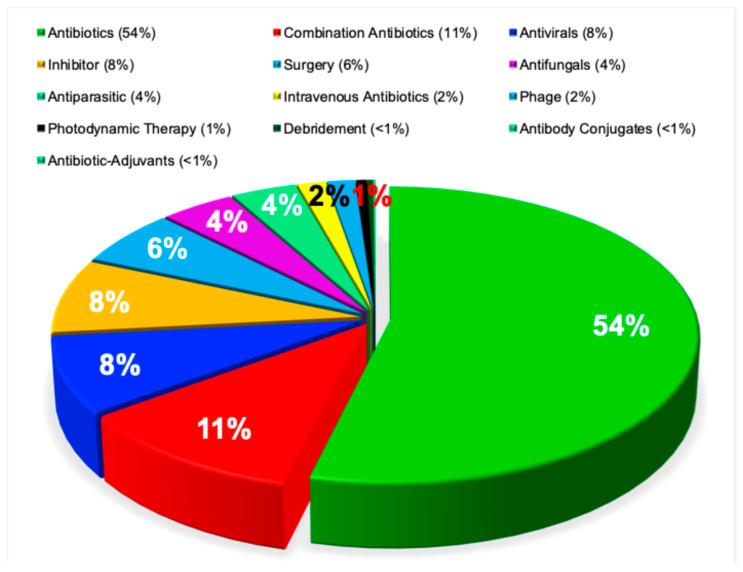
Research into treatments for antimicrobial resistance. This pie chart illustrates the distribution of 191,999 research publications on treatments targeting antimicrobial resistance, categorized by antimicrobial agents, antiviral therapies, and resistance inhibitors.

In contrast to these disparate reports [59,60,61,62], the present bibliometric analysis reveals a clear and statistically distinct distribution of treatment strategies for antimicrobial resistance. The use of newer, more potent antibiotics was the most frequently reported approach, accounting for 54% of cases. This was followed by combination antibiotic therapy (11%), antiviral agents (8%), resistance inhibitors (8%), surgical interventions (6%), antifungal therapies (4%), and antiparasitic drugs (4%). Other modalities, including intravenous antibiotic escalation, bacteriophage therapy, photodynamic therapy, debridement, antibody conjugates, and antibody adjuvants, were each reported in fewer than 2% of cases. The overall treatment distribution differed markedly from uniformity and was highly statistically significant (χ^2^ = 616,314, *p* < 0.0001), as shown in Figure 7.

### 3.8. Antimicrobial Resistance Research by Pathogen Type

Viruses [63], bacteria [64], fungi [65], and parasites [66] have all been identified as the major pathogen global threats to human morbidity, disability, and mortality.

**Figure 8:**
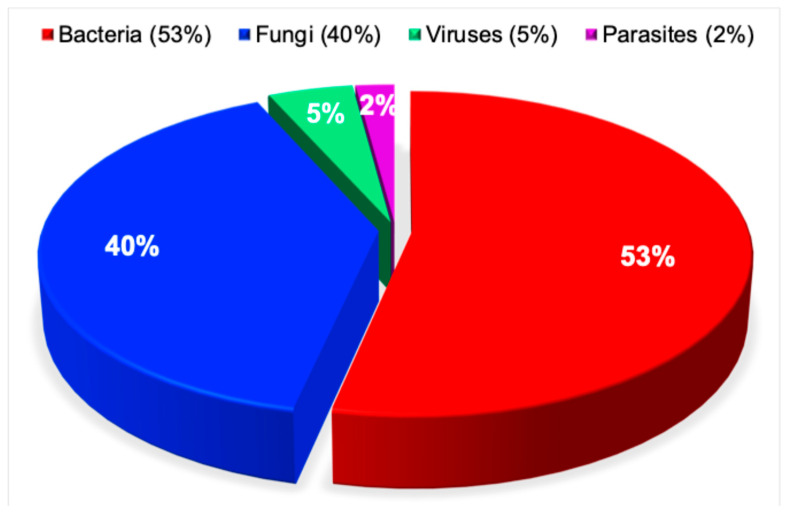
Antimicrobial resistance research by pathogen type. This pie chart depicts the distribution of 83,593 research publications on antimicrobial resistance, categorized by pathogen type, including bacteria, fungi, viruses, and parasites.

In contrast to these broad epidemiological assessments [63,64,65,66], the present analysis reveals a distinct distribution among pathogens specifically associated with antimicrobial resistance. Bacteria accounted for the majority of reported antimicrobial resistance studies at 53%, followed by fungi at 40%, viruses at 5%, and parasites at 2%, highlighting the disproportionate concentration of research activity and reported resistance burden in bacterial and fungal pathogens relative to viral and parasitic organisms. While this pattern may partly reflect true differences in resistance prevalence and clinical impact, it may also be influenced by differential research prioritization and funding allocation across pathogen categories. This highly statistically significant disparity (χ^2^ = 66,089, *p* < 0.0001) is illustrated in Figure 8.

### 3.9. Antimicrobial Resistance Research Involving Superbugs

Superbug is a non-specific term used to describe strains of bacteria that are resistant to at least one or more commonly used antibiotics [67]. These pathogens include Methicillin-resistant *Staphylococcus aureus* (MRSA) [68], *Clostridioides difficile* (*C. diff*) [69], drug-resistant Neisseria gonorrhoeae (DRG) [70], multidrug-resistant *Mycobacterium tuberculosis* (MDR TB) [71], vancomycin-resistant Enterococcus (VRE) [72], carbapenem-resistant *Enterobacterales* (CRE) [73], *Pseudomonas aeruginosa* (*P. aeruginosa*) [74], *Acinetobacter baumannii* (*A. baumannii*) [75], *Streptococcus pneumoniae* associated with “pneumococcal disease” [76], and *Bordetella pertussis*, the causative agent of whooping cough [77].

**Figure 9:**
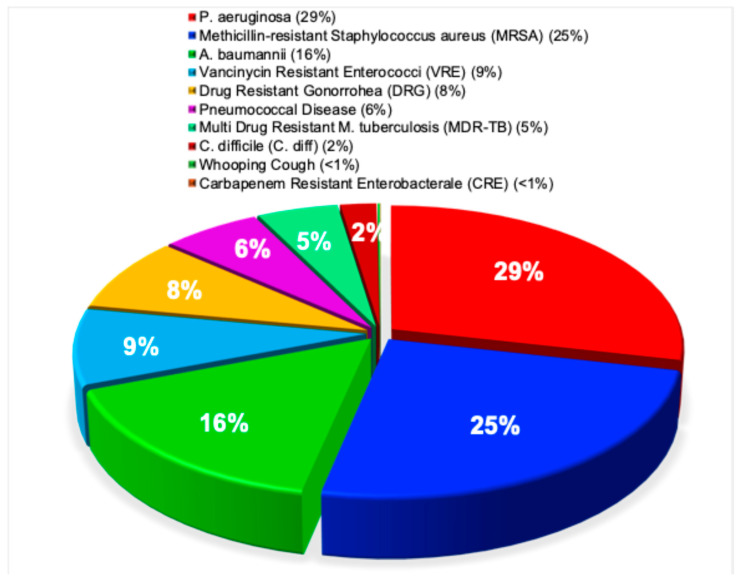
Antimicrobial resistance research involving superbugs. This pie chart illustrates the distribution of 16,784 antimicrobial resistance publications, highlighting the relative research focus on the major “superbugs” *P. aeruginosa*, MRSA, and *A. baumannii*, VRE, DRG, pneumococcal disease, MDR-TB, *C. diff.*, whooping cough, and CRE.

In contrast to the heterogeneous emphasis across prior reports [67,68,69,70,71,72,73,74,75,76,77], the present analysis reveals a distinct distribution of superbug-related burden. More than half of reported cases were attributable to *P. aeruginosa* (29%) and MRSA (25%), highlighting their dominant role among resistant pathogens. *A. baumannii* accounted for a further 16% of reports. All remaining superbugs, including VRE, DRG, pneumococcal disease, MDR TB, *C. diff*, whooping cough, and CRE, each comprised fewer than 9% of reported cases. This distribution was highly statistically significant (χ^2^ = 15,068, *p* < 0.0001), as illustrated in Figure 9.

### 3.10. Mechanisms of Antibiotic Resistance in Pathogenic Bacteria

**Figure 10:**
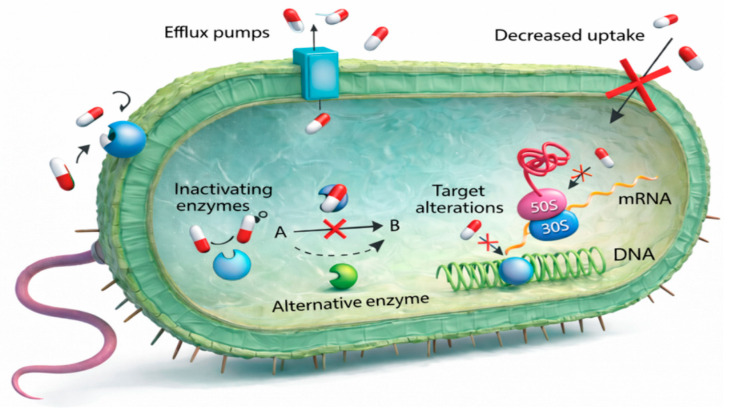
Mechanisms of antibiotic resistance in pathogenic bacteria. This illustration presents the core mechanisms by which pathogenic bacteria evade antibiotic activity, including reduced drug uptake, active efflux via membrane pumps, enzymatic drug inactivation, and structural modification of antibiotic targets.

Pathogenic bacteria employ a diverse array of mechanisms to evade the effects of antibiotics. These include genetic mutations that alter drug targets or regulatory pathways [78], reduced antibiotic uptake through changes in membrane permeability [79], active efflux systems that expel antibiotics from the cell [80], utilization of alternative enzymatic pathways [81], receptor or target site modifications [82], enzymatic inactivation of antibiotics [83], and interference with mRNA, DNA, or protein synthesis [84]. Collectively, these resistance mechanisms also represent critical therapeutic targets for the development of strategies aimed at restoring antimicrobial efficacy or preventing resistance emergence [85], as illustrated from a drawing and rendered in three-dimensions using OpenAI (https://openai.com/, accessed on 9 January 2025) (San Francisco, CA, USA) in Figure 10.

**Figure 11:**
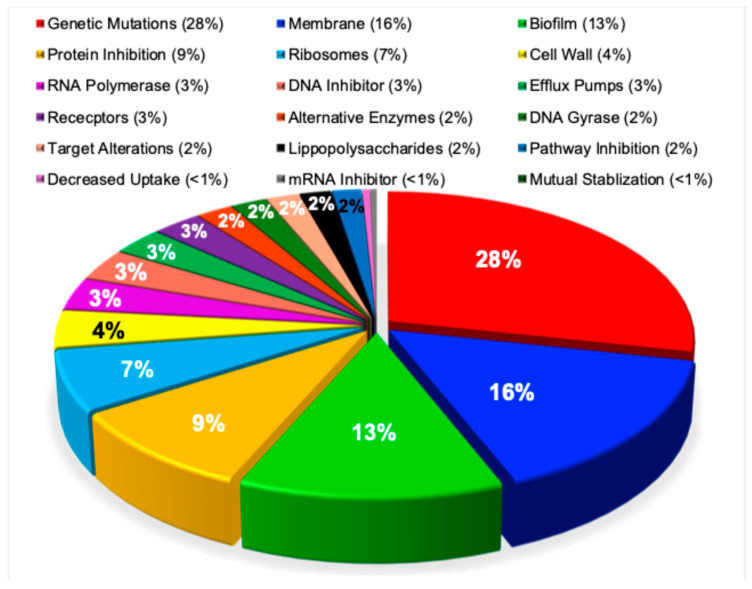
Research into the antibiotic resistance mechanisms of pathogenic bacteria. This pie chart displays the distribution of 123,627 research publications examining resistance mechanisms in pathogenic bacteria, including genetic mutations, membranes, protein inhibition, ribosomes, cell wall, and RNA polymerase.

In contrast to the heterogeneous emphasis placed on resistance mechanisms across prior studies [78,79,80,81,82,83,84], the present bibliometric analysis reveals a clearly defined and nonuniform distribution in the reporting of resistance pathways. Genetic mutations accounted for nearly one-third of reported cases (28%), underscoring their central role in resistance development. This was followed by membrane-associated mechanisms (16%), biofilm (13%), protein inhibition pathways (9%), and ribosomal alterations (7%), highlighting these systems as dominant targets for antimicrobial intervention. Mechanisms involving the cell wall, RNA polymerase, DNA inhibition, efflux pumps, and receptor-mediated resistance each accounted for approximately 3–4% of reports. Alternative enzymatic pathways, DNA gyrase modifications, target alterations, lipopolysaccharide-mediated resistance, and pathway inhibition were each reported in approximately 2% of cases. The least frequently reported mechanisms included decreased antibiotic uptake, mRNA inhibition, mutual stabilization, and sequential blockade strategies, each representing less than 1% of the literature. This distribution differed markedly from uniformity and was highly statistically significant (χ^2^ = 208,980, *p* < 0.0001), as shown in Figure 11.

### 3.11. Thematic Map of Antimicrobial Resistance

**Figure 12:**
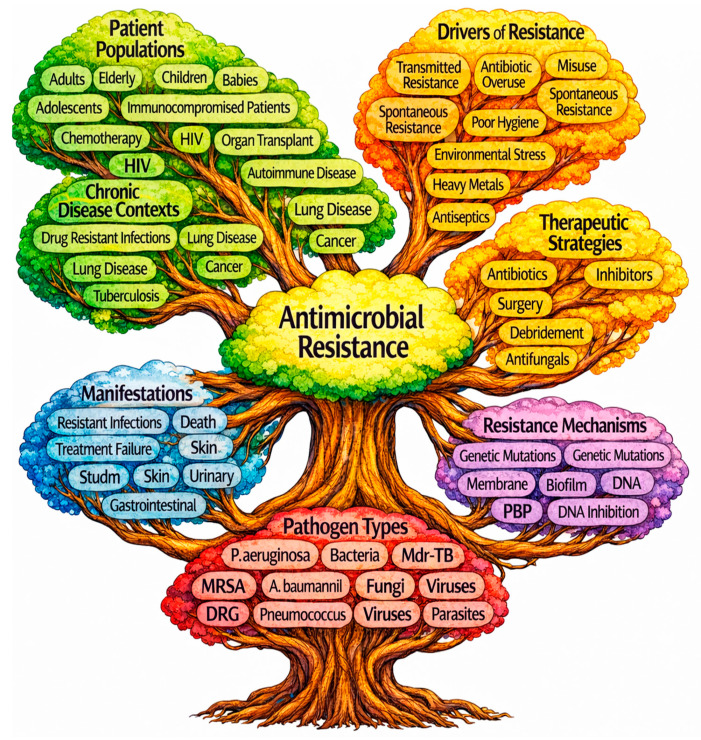
Thematic landscape of antimicrobial resistance research publications. This thematic landscape synthesizes the principal domains of antimicrobial resistance research, encompassing affected patient populations, drivers of resistance, clinical manifestations, therapeutic strategies, underlying resistance mechanisms, and key pathogen types.

A thematic map of antimicrobial resistance can be organized around a central core with six major interconnected domains. At the center, antimicrobial resistance links to major research clusters encompassing patient populations, drivers of resistance, clinical manifestations, therapeutic strategies, pathogen types, and resistance mechanisms. Patient-focused themes emphasize age groups, immunocompromised states, and chronic disease contexts, while drivers of resistance highlight transmitted resistance, antibiotic overuse and misuse, and environmental contributors. Clinical manifestations cluster around resistant infections, organ-specific syndromes, and severe outcomes. Therapeutic strategies remain predominantly antibiotic-centered, with adjunctive and alternative approaches forming secondary branches. Pathogen-related themes are dominated by bacterial and fungal resistance, with priority organisms, such as multidrug-resistant bacteria, receiving focused attention. Mechanistic clusters emphasize genetic mutations, membrane-associated processes, biofilms, and target-based resistance pathways. Together, the mind map shown in Figure 12 illustrates a patient-centered, pathogen-driven, and mechanism-intensive research landscape in antimicrobial resistance.

### 3.12. Summary of Key Mechanisms, Drivers, and Strategies for Combating Antibiotic Resistance

The interactions among resistance mechanisms, selective drivers, and therapeutic strategies within the oral, gut, and skin microbiota are highly complex and deeply interconnected. Understanding these relationships is essential for the rational design of effective interventions against antimicrobial-resistant oral infections. Resistance within the oral microbiome arises from the convergence of microbial genetic adaptation, environmental and clinical drivers of antimicrobial exposure, and the unique ecological dynamics of oral biofilms that facilitate persistence, gene exchange, and tolerance to therapy.

Effective mitigation, therefore, requires coordinated, multifaceted strategies that integrate mechanistic insights with prevention, targeted treatment, antimicrobial stewardship, and ongoing surveillance. The relationships among the principal resistance mechanisms, key drivers of antimicrobial resistance, associated genetic determinants, affected oral pathogens, and emerging or alternative therapeutic approaches are shown in Table 1.

Together, these elements highlight critical leverage points for intervention and provide a structured framework for strategies aimed at limiting the development and spread of antibiotic resistance within the oral microbiota and other pathogens.

## 4. Discussion

This study represents the first comprehensive bibliometric assessment integrating antimicrobial resistance research across pathogens, patient-level factors, resistance mechanisms, and therapeutic strategies within the biomedical literature, according to the authors’ knowledge. By examining these dimensions together rather than in isolation, the analysis reveals a research landscape that is internally coherent yet uneven, shaped as much by biological realities as by structural priorities in global health research. The findings expose critical asymmetries in how antimicrobial resistance is studied, framed, and addressed, with important implications for prevention, diagnosis, and treatment.

### 4.1. Patient Factors and Disparities in Antimicrobial Resistance Burden

One of the most striking findings is the uneven distribution of antimicrobial resistance across patient age groups and immunocompromised conditions. Adults and older patients together accounted for the majority of reported resistant infections, whereas infants and children were comparatively underrepresented. This pattern likely reflects cumulative antimicrobial exposure, higher rates of hospitalization, and the increasing prevalence of chronic disease with age [94]. At the same time, it raises concern that pediatric resistance burdens may be under-characterized, particularly in low-resource settings where surveillance infrastructure is limited and infections may be underreported.

Among immunocompromised populations, patients receiving chemotherapy overwhelmingly dominated resistance-related reports. This finding aligns with established clinical vulnerabilities, including prolonged neutropenia, frequent healthcare contact, and repeated exposure to broad-spectrum antimicrobials [95]. However, the comparatively lower representation of individuals with HIV AIDS, transplant recipients, and autoimmune disease suggests that research emphasis may not accurately reflect global disease prevalence or cumulative risk [96]. These disparities highlight potential blind spots in surveillance and reinforce the need for broader inclusion of diverse immunocompromised populations in antimicrobial resistance research and clinical trials.

### 4.2. Drivers of Resistance Emphasize Transmission over Individual Misuse

Perhaps the most consequential finding of this review is the identification of transmitted resistance as the dominant driver of antimicrobial resistance, exceeding antibiotic overuse and misuse. This observation challenges a long-standing narrative that frames resistance primarily as a consequence of individual prescribing behavior [97]. Instead, it underscores the central role of transmission dynamics, including healthcare-associated spread, environmental reservoirs, and population-level circulation of resistant organisms.

The prominence of transmitted resistance reframes antimicrobial resistance as a collective systems problem rather than a series of isolated treatment failures. This shift carries major implications for prevention strategies, indicating that antimicrobial stewardship must be coupled with robust infection control, environmental decontamination, cohorting practices, and active surveillance [98]. Interventions that focus narrowly on reducing prescriptions without addressing transmission pathways are unlikely to achieve sustained impact.

### 4.3. Clinical Presentation Reflects Diagnostic Limitations

The predominance of resistant infection itself as the most frequently reported clinical manifestation reflects persistent limitations in how antimicrobial resistance is detected and documented. Most studies rely on phenotypic treatment failure or culture-based resistance rather than early molecular or genomic markers. This reactive diagnostic approach delays targeted intervention [99], prolongs empirical therapy, and further amplifies selective pressure.

The relatively low reporting of systemic complications such as cardiovascular events, gangrene, or impaired wound healing should not be interpreted as evidence of rarity. Rather, it reflects how resistance is framed in the literature, often as an endpoint rather than as a driver of downstream morbidity, disability, and healthcare utilization, the so-called “disaster research” [100]. This narrow framing likely underestimates the true clinical and economic burden of antimicrobial resistance.

### 4.4. Therapeutic Strategies Remain Conservative Despite Expanding Knowledge

Despite decades of research elucidating resistance mechanisms, therapeutic responses remain largely conservative. Escalation to newer or more potent antibiotics continues to dominate clinical management, while combination therapies, inhibitors, and alternative approaches, such as bacteriophages or photodynamic therapy, remain comparatively remembered. This disconnect suggests a persistent gap between mechanistic understanding and clinical implementation.

The limited adoption of combination therapies is particularly notable given the high prevalence of multidrug resistance mechanisms identified in this review. Strategies that exploit synergistic interactions, parallel pathway inhibition, or resistance suppression offer clear theoretical advantages, yet they remain underrepresented in practice [101]. This gap likely reflects regulatory hurdles, limited randomized trial data, and concerns regarding toxicity and complexity rather than a lack of biological rationale.

### 4.5. Pathogen Focus Reveals Structural Research Biases

The dominance of bacterial pathogens, particularly *P. aeruginosa* and MRSA, reflects both their clinical severity and the concentration of research infrastructure around hospital-associated infections [102]. While this focus is justified by disease burden, it may inadvertently marginalize fungal and parasitic resistance, which comprises a substantial minority of reports yet remains underrepresented in therapeutic innovation.

The prominence of *P. aeruginosa* over MRSA is particularly noteworthy. Although MRSA remains a highly feared pathogen and a widely recognized threat, it is *P. aeruginosa’s* intrinsic resistance, metabolic flexibility, and environmental persistence that may position it as a more formidable long-term threat [103]. This finding supports calls to expand research investment beyond historically prioritized organisms.

### 4.6. Resistance Mechanisms Highlight Translational Gaps

Genetic mutations, membrane-associated mechanisms, biofilm formation, and protein synthesis inhibition dominated reported resistance pathways, consistent with decades of molecular research [104]. While these findings validate existing mechanistic frameworks, the near absence of higher-order strategies, such as sequential blockade and mutual stabilization, underscores a translational gap.

This imbalance suggests that resistance mechanisms are being extensively cataloged but insufficiently leveraged to inform therapeutic design [105]. Progress in the field will require a shift from descriptive mechanistic studies toward intervention-focused research that directly tests how resistance pathways can be disrupted in real-world clinical settings.

### 4.7. Implications for the Oral Microbiota

The oral microbiota represents a particularly complex and underappreciated reservoir for antimicrobial resistance [106]. Dense polymicrobial biofilms, frequent antimicrobial exposure, and extensive horizontal gene transfer create ideal conditions for resistance emergence and persistence [107]. The integrative framework presented in Table 1 illustrates how resistance mechanisms, drivers, pathogens, and therapies intersect in this niche and highlights opportunities for targeted intervention.

Precision therapies, localized drug delivery systems, antimicrobial peptides, and stewardship strategies tailored to dental and medical practices may offer disproportionate benefits by limiting resistance amplification at a critical interface between healthcare and the community.

### 4.8. Limitations of Bibliometric Research

Bibliometric research has inherent database limitations, as it describes patterns of publication rather than scientific quality, clinical relevance, or methodological rigor. Findings are influenced by database coverage, indexing practices, and terminology variability, which may lead to incomplete representation of the literature. Keyword frequency and co-occurrence analyses reflect associations in how topics are discussed, not causal or biological relationships. In addition, publication bias and time-dependent indexing effects may overrepresent well-studied or well-funded topics while underrepresenting emerging or less visible areas.

### 4.9. Future Research Directions

To enhance the clinical relevance of patient-level factors in antimicrobial resistance, the recent literature emphasizes the importance of early risk assessment for colonization and infection with multidrug-resistant pathogens as a means to inform screening strategies, infection control practices, and empiric antimicrobial selection [108]. Contemporary studies demonstrate that integrating readily available clinical variables, including prior antibiotic exposure, healthcare contact, comorbidity burden, and device use, can improve identification of patients at highest risk, although predictive performance remains heterogeneous and context dependent [109]. Beyond clinical predictors alone, emerging personalized medicine approaches provide an opportunity to strengthen translational relevance. Recent work has explored host response biomarkers, such as microRNA-related profiling and circulating plasma gelsolin, as potential tools for refining individual susceptibility assessment and prognostication. Incorporating biomarker-based stratification alongside traditional clinical risk factors may support more precise, patient-tailored antimicrobial stewardship and earlier intervention in multidrug-resistant infections.

## 5. Conclusions and Future Directions

This bibliometric review synthesizes how antimicrobial resistance is represented in MEDLINE-indexed articles across patient populations, pathogens, resistance mechanisms, and therapeutic strategies. The findings indicate that antimicrobial resistance is shaped not only by microbial evolution but also by transmission dynamics, healthcare practices, patient vulnerabilities, and prevailing research priorities.

Clear trends emerge from the data. Adults and patients undergoing chemotherapy account for a disproportionate share of reported resistant infections, while transmitted resistance is consistently identified as the dominant driver of acquisition. Research attention remains heavily concentrated on bacterial pathogens and genetic mechanisms of resistance, with comparatively less emphasis on fungal resistance, host factors, and higher-order therapeutic strategies. Despite substantial advances in mechanistic understanding, clinical management in the literature continues to rely predominantly on antibiotic escalation, with limited integration of combination approaches, adjunctive therapies, or early risk stratification frameworks.

These patterns reveal important gaps between mechanistic knowledge and translational clinical applications. Underrepresentation of fungal and non-bacterial resistance, limited focus on patient-level risk profiling, and minimal incorporation of personalized or biomarker-informed approaches suggest that the field has advanced more rapidly in description than in implementation. Bridging this divide requires a shift from reactive treatment models toward integrated prevention, early detection, and mechanism-informed therapeutic strategies that align microbiological insights with patient-specific vulnerability.

Future studies should, therefore, combine bibliometric insights with qualitative, experimental, and clinical research to better connect publication trends with biological relevance, therapeutic innovation, and measurable patient outcomes.

## Figures and Tables

**Table 1:** Summary of key mechanisms, drivers, and strategies for combating antibiotic resistance in the oral microbiota.

Aspect	Key Details and Examples	Clinical Implications
Major resistance mechanisms [86,87]	Efflux pumps (e.g., *P*. *gingivalis*)	Reduced intracellular drug concentrations leading to diminished efficacy of antibiotics and antifungals
	Enzymatic inactivation (e.g., β-lactamases)	Persistent, chronic, and recurrent oral infections
	Target modification (e.g., *erm* genes conferring macrolide resistance)	Failure of first-line therapies and need for alternative agents
	Biofilm formation (e.g., *C*. *albicans*)	Enhanced tolerance to antimicrobials and host defenses
Drivers of antimicrobial resistance [88,89]	Overuse and misuse of antibiotics in dental practice	Increased prevalence of multidrug-resistant oral pathogens
	Exposure to antiseptics, biocides, and heavy metals	Co-selection and maintenance of resistance genes
	Horizontal gene transfer within polymicrobial biofilms	Rapid dissemination of resistance traits across species
AMR-associated genes [90]	*tetM* (tetracycline resistance)	Detected in both healthy and diseased oral microbiota
	*ermB*, *mefA/E* (macrolide resistance)	High abundance in supra-gingival biofilms and saliva
	*blaZ*, *cfxA* (β-lactam resistance)	Reduced effectiveness of penicillins and cephalosporins
Affected pathogens [91]	*P*. *gingivalis*, *Prevotella* spp. *F*. *nucleatum*, and *A*. *actinomycetemcomitans*	Resistance to amoxicillin, clindamycin, metronidazole, tetracycline, erythromycin, and other antibiotics
	*Candida* spp.	Reduced susceptibility to azole and polyene antifungals
Alternative and adjunctive therapies [92]	Antimicrobial photodynamic therapy (aPDT)	Reduces reliance on broad-spectrum antibiotics
	Antimicrobial peptides (AMPs)	Target pathogens with limited disruption of commensal microbiota
	Probiotics and prebiotics	Modulate oral microbial ecology and suppress resistant strains
	Targeted delivery systems (e.g., nanoparticles, microspheres)	Enhanced local drug concentration with reduced systemic exposure
	Precision-guided peptides (e.g., C16G2)	Selective elimination of pathogenic species
Stewardship and surveillance strategies [93]	Education and training of dental professionals	Promotes rational and evidence-based prescribing
	Implementation of clinical guidelines	Enables early detection and targeted intervention
	Use of diagnostic tools (molecular, metagenomic)	Improves pathogen identification and resistance profiling
	Oral-specific antimicrobial resistance surveillance programs	Monitors emerging resistance trends and informs policy

## Data Availability

The published data is available from the articles referenced. The raw data for each of the analyses are shown in the Supplemental Data File.

## Supplementary Materials

The following supporting information can be downloaded at: https://www.mdpi.com/article/10.3390/pathogens15030288/s1, Figure S1. Number of Publications on Antimicrobial Resistance; Figure S2. Patients “antimicrobial resistance” in pubmed and keywords; Figure S3. “immunocompromised patients antimicrobial resistance” in pubmed and keywords; Figure S4. “patients chronic antimicrobial resistance” in pubmed and keywords; Figure S5. Reasons for “patients antimicrobial resistance” in pubmed and keywords; Figure S6. “patients symptoms antimicrobial resistance” in Pubmed and keywords; Figure S7. “treatments antimicrobial resistance” in Pubmed and keywords; Figure S8. “Types antimicrobial resistance” in Pubmed and keywords; Figure S9. “superbugs antimicrobial resistance” in Pubmed and keywords; Figure S10. Mechanisms of antibiotic resistance” in Pubmed and keywords.
